# Crosstalk between Smad2/3 and specific isoforms of ERK in TGF‐β1‐induced TIMP‐3 expression in rat chondrocytes

**DOI:** 10.1111/jcmm.13099

**Published:** 2017-02-23

**Authors:** Yanhui Zhu, Jianhua Gu, Tong Zhu, Chen Jin, Xiaopeng Hu, Xiang Wang

**Affiliations:** ^1^ Shanghai Key Laboratory of Orthopaedic Implants Department of Orthopaedic Surgery Shanghai Ninth People's Hospital Shanghai JiaoTong University School of Medicine Shanghai China

**Keywords:** Osteoarthritis, TGF‐β1, TIMP3, Crosstalk, Smad2/3, ERK isoforms

## Abstract

This study investigated the roles of ERK1 and ERK2 in transforming growth factor‐β1 (TGF‐β1)‐induced tissue inhibitor of metalloproteinases‐3 (TIMP‐3) expression in rat chondrocytes, and the specific roles of ERK1 and ERK2 in crosstalk with Smad2/3 were investigated to demonstrate the molecular mechanism of ERK1/2 regulation of TGF‐β1 signalling. To examine the interaction of specific isoforms of ERK and the Smad2/3 signalling pathway, chondrocytes were infected with LV expressing either ERK1 or ERK2 siRNA and stimulated with or without TGF‐β1. At indicated time‐points, TIMP‐3 expression was determined by real‐time PCR and Western blotting; p‐Smad3, nuclear p‐Smad3, Smad2/3, p‐ERK1/2 and ERK1/2 levels were assessed. And then, aggrecan, type II collagen and the intensity of matrix were examined. TGF‐β1‐induced TIMP‐3 expression was significantly inhibited by ERK1 knock‐down, and the decrease in TIMP‐3 expression was accompanied by a reduction of p‐Smad3 in ERK1 knock‐down cells. Knock‐down of ERK2 had no effect on neither TGF‐β1‐induced TIMP‐3 expression nor the quantity of p‐Smad3. Moreover, aggrecan, type II collagen expression and the intensity of matrix were significantly suppressed by ERK1 knock‐down instead of ERK2 knock‐down. Taken together, ERK1 and ERK2 have different roles in TGF‐β1‐induced TIMP‐3 expression in rat chondrocytes. ERK1 instead of ERK2 can regulate TGF‐β/Smad signalling, which may be the mechanism through which ERK1 regulates TGF‐β1‐induced TIMP‐3 expression.

## Introduction

During the pathology of osteoarthritis, chondrocytes synthesize matrix metalloproteinases (MMPs), which can collectively digest major cartilage extracellular matrix (ECM) components. Transforming growth factor‐β1 (TGF‐β1) plays an important role in the control of both cartilage homoeostasis and repair ,which has been extensively explored over the recent years. These studies have established that TGF‐β1 stimulates the expression of tissue inhibitors of metalloproteinases (TIMPs), as natural inhibitors of MMPs. Therefore, TGF‐β1‐induced TIMPs expression represents a key regulatory point in osteoarthritis progression.

Smad2/3,essential components of the intracellular TGF‐β1 signalling pathway, participates in TIMPs expression, which may play a role in ECM homoeostasis, tissue repair and fibrosis 1, 2, 3. It has been reported that Smad2/3 signalling is a critical mediator of TGF‐β‐induced TIMP‐3 expression in human chondrocytes, and the TIMP‐3 gene is a target of Smad signalling 4. In addition to TGF‐β/Smad signalling, other intracellular signalling molecules, such as extracellular signal‐regulated kinase1/kinase2 (ERK1/2), are important regulators of TIMPs expression 5, 6. Further studies have indicated that TGF‐β‐induced TIMP‐1 expression is promoted via activation of the ERK1/2 pathway and Sp1 transcription factor in human fibrosarcoma cells 7. Similarly, the ERK1/2 signalling pathway and Sp1 transcription factor play a pivotal role in regulating TGF‐β‐induced TIMP‐3 expression in chondrocytes 8. Recently, we have demonstrated that both the Smad2/3 and ERK1/2 signalling pathways are involved in TGF‐β1‐induced TIMP‐3 expression, and ERK1/2 may be capable of activating the Smad2/3 signalling pathway to result in the TGF‐β1‐induced TIMP‐3 up‐regulation 9. However, the respective roles of ERK1 and ERK2 in TGF‐β1‐induced TIMP‐3 expression, and their functional interactions with Smad2/3 have not been established in rat chondrocytes.

Herein, we synthesized and screened specific and efficient LV expressing siRNAs for ERK1 and ERK2 silencing. Either ERK1 or ERK2 was silenced in rat chondrocytes to investigate the roles of ERK1 and ERK2 in crosstalk with Smad2/3 signalling in relation to TGF‐β1‐induced TIMP‐3 expression.

## Materials and methods

### Chondrocyte isolation

Normal rat knee cartilage was obtained from the tibial platform and the femoral condyle, in accordance with protocols approved by the Shanghai Jiao Tong University School of Medicine Ethics Committee. Cartilage pieces were washed twice with phosphate‐buffered saline (PBS) and treated with 0.2 mg/ml collagenase (Sigma Chemical Co., Poole, UK) in serum‐free Dulbecco's modified Eagle's medium (DMEM) overnight at 37°C. The cells were collected by filtering through a 200‐μm mesh nylon cell strainer, centrifuged at 1000 × *g* for 5 min. and washed twice with PBS. Finally, the cells were resuspended and cultured in DMEM supplemented with 10% (vol/vol) foetal bovine serum (FBS) (Invitrogen, Carlsbad, CA, USA), plus 1% penicillin and streptomycin (GIBCO‐BRL, San Diego, CA, USA). The culture medium was changed every other day. The chondrocytic phenotype of the cultured cells was confirmed by positive immunostaining for type II collagen and toluidine blue staining of glycosaminoglycans. First passage chondrocytes were used in all experiments.

### Design of ERK1 and ERK2 siRNAs

The rat ERK1‐ and ERK2‐specific siRNAs were screened and selected based on NCBI reference sequences (GenBank: ERK1: NM_017347 and ERK2: NM_053842). Three siRNA oligomers were chosen to target the ERK1 and ERK2 coding sequences, respectively, and a negative siRNA served as a control. The sequences of the siRNA oligomers and the corresponding oligonucleotide sequences, designated ERK1 siRNA1, 2, 3, ERK2 siRNA1, 2, 3 and negative siRNA, are shown in Table 1. Then, the corresponding oligonucleotide sequences for the ERK1, ERK2 and negative siRNAs were synthesized (Invitrogen), annealed and subcloned into pMAGic 7.1 (CMV‐GFP‐T2A‐Puro).

**Table 1:** siRNA oligomer sequences and oligonucleotide sequences

siRNA	siRNA oligomers (5′‐3′)	Oligonucleotide sequences (5′‐3′)
ERK1 siRNA1	GGACCTGTACAAGCTGCTA	Forward:CCGGGGACCTGTACAAGCTGCTATTCAAGAGATAGCAGCTTGTACAGGTCCTTTTTTG
		Reverse:AATTCAAAAAATGGACCTGTACAAGCTGCTATCTCTTGAATAGCAGCTTGTACAGGTCC
ERK1 siRNA2	GGACCAGCTCAACCACATT	Forward:CCGGGGACCAGCTCAACCACATTATCAAGAGTAATGTGGTTGAGCTGGTCCTTTTTTG
		Reverse:AATTCAAAAAATGGACCAGCTCAACCACATTACTCTTGATAATGTGGTTGAGCTGGTCC
ERK1 siRNA3	GCGCATCACAGTAGAGGAA	Forward:CCGGGCGCATCACAGTAGAGGAATTCAAGAGATTCCTCTACTGTGATGCGCTTTTTTG
		Reverse:AATTCAAAAAATGCGCATCACAGTAGAGGAATCTCTTGAATTCCTCTACTGTGATGCGC
ERK2 siRNA1	GCAATGATCATATCTGCTA	Forward:CCGGGCAATGATCATATCTGCTATTCAAGAGATAGCAGATATGATCATTGCTTTTTTG
		Reverse:AATTCAAAAAATGCAATGATCATATCTGCTATCTCTTGAATAGCAGATATGATCATTGC
ERK2 siRNA2	GGTATTCTTGGATCTCCAT	Forward:CCGGGGTATTCTTGGATCTCCATTTCAAGAGAATGGAGATCCAAGAATACCTTTTTTG
		Reverse:AATTCAAAAAATGGTATTCTTGGATCTCCATTCTCTTGAAATGGAGATCCAAGAATACC
ERK2 siRNA3	GGACCTCATGGAGACAGAT	Forward:CCGGGGACCTCATGGAGACAGATATCAAGAGAATCTGTCTCCATGAGGTCCTTTTTTG
		Reverse:AATTCAAAAAATGGACCTCATGGAGACAGATTCTCTTGATATCTGTCTCCATGAGGTCC
Negative siRNA	TTCTCCGAACGTGTCACGT	Forward:CCGGTTCTCCGAACGTGTCACGTTTCAAGAGAACGTGACACGTTCGGAGAATTTTTG
		Reverse:AATTCAAAAATTCTCCGAACGTGTCACGTTCTCTTGAAACGTGACACGTTCGGAGAA

The pPACK Packaging Plasmid Mix (System Biosciences, Palo Alto, CA, USA) and lentiviral transfer vectors containing the seven siRNA sequences were cotransfected into 293 cells using Lipofectamine™ 2000 transfection reagent (Invitrogen). After 24‐hrs incubation, the transfection solution was removed and the medium was changed to DMEM containing 1% FBS. Lentiviral supernatant was collected after 48 hrs of incubation, and cellular debris was eliminated by centrifugation at 5000 × *g* for 5 min. Subsequently, the lentiviral supernatant was filtered through 0.45 μm polyvinylidene fluoride (PVDF) filters (Millipore, Watford, UK). The titres of LV expressing the seven siRNAs were measured by infecting 293 cells with serial dilutions of concentrated LV. The lentiviral supernatant was adjusted to 1 × 10^4^ ifu/ml using Dulbecco's PBS.

First passage chondrocytes were seeded into 6‐mm dishes and cultured in a humidified incubator at 37°C with 5% CO_2_. The chondrocytes were infected with LV expressing the seven siRNAs at an optimum multiplicity of infection (MOI) of 20 when the cells reached 50% confluence; for controls, cells were left untreated. Real‐time PCR 96 hrs after infection revealed that ERK1 siRNA2 and ERK2 siRNA2 were the most efficient siRNAs for ERK1 and ERK2 silencing. To test the efficiency of these siRNAs at suppressing ERK1 and ERK2 protein expression, chondrocytes were infected with LV expressing ERK1 siRNA2 and ERK2 siRNA2, and cell lysate was collected 96 hrs after infection. And ERK1 and ERK2 protein was determined by Western blotting. To examine the specificity of the siRNAs, ERK1 siRNA2 against ERK2 expression and ERK2 siRNA2 against ERK1 expression were determined by real‐time PCR and Western blotting.

### Chondrocyte infection and treatments

To analyse the involvement of the ERK1/2, ERK1, ERK2 and Smad2/3 signalling pathways in TGF‐β1‐induced TIMP‐3 expression, first passage chondrocytes were seeded into six‐well plates (3 × 10^5^ cells/well) and cultured in a humidified incubator at 37°C with 5% CO_2_. The chondrocytes were infected with LV expressing ERK1 siRNA2/ERK2 siRNA2 at an MOI of 20 for 96 hrs when the cells reached 70–80% confluence. The cells were then stimulated with or without 10 ng/ml TGF‐β1 for 48 hrs. TIMP‐3 expression was evaluated by real‐time PCR and Western blotting. Additionally, the chondrocytes were infected with LV expressing ERK1 siRNA2/ERK2 siRNA2/not infected. The cells were stimulated with 10 ng/ml TGF‐β1. P‐Smad3, Smad2/3, p‐ERK1/2 and ERK1/2 levels were examined by Western blotting at 0‐, 10‐, 15‐, 30‐ and 60‐min. time‐points.

To examine the interaction between specific isoforms of the ERK and Smad2/3 signalling pathways, chondrocytes were infected with or without LV expressing the ERK1 siRNA2/ERK2 siRNA2 for 96 hrs and then stimulated with 10 ng/ml of TGF‐β1 for 15 and 30 min. The levels of p‐Smad3, Smad2/3, p‐ERK1/2 and ERK1/2 expression were examined by Western blotting. And then, we examined the target genes of TGF‐β‐Smad2/3 signalling, such as aggrecan and type II collagen expression by ERK1/ERK2 knock‐down. The chondrocytes were infected with LV expressing ERK1 siRNA2/ERK2 siRNA2 for 96 hrs and then, stimulated with or without 10 ng/ml TGF‐β1 for 48 hrs. Aggrecan and type II collagen expression was evaluated by real‐time PCR and Western blotting. Additionally, first passage chondrocytes were seeded into 24‐well plates (1 × 10^5^ cells/well) which were infected with LV expressing ERK1 siRNA2/ERK2 siRNA2 for 96 hrs and then, stimulated with or without 10 ng/ml TGF‐β1 for 48 hrs. Toluidine blue staining of matrix was performed.

To further examine the accumulation of nuclear p‐Smad3 following knock‐down of ERK1 or ERK2, chondrocytes were infected with or without LV expressing the ERK1 siRNA2/ERK2 siRNA2 for 96 hrs and then stimulated with 10 ng/ml of TGF‐β1 for 30 min., and nuclear p‐Smad3 levels were examined by Western blotting.

### RNA extraction and real‐time PCR

Total RNAs were isolated using TRIZOL reagent (Invitrogen) according to the manufacturer's protocol. For first‐strand cDNA synthesis, 2 μg total RNA was reverse transcribed using the Reverse Transcriptase Moloney murine leukaemia virus (M‐MLV) cDNA Synthesis kit (Takara, Tokyo, Japan) according to the manufacturer's protocol. Briefly, a 12‐μl reaction mixture containing 2 μl oligo d(T)18 primer (50 μM), 2 μg total RNA and RNAse‐free dH_2_O was incubated at 70°C for 10 min. Subsequently, 1 μl deoxynucleotide triphosphate mixture (10 mM each), 4 μl 5× M‐MLV buffer, 0.5 μl ribonuclease inhibitor (40 U/μl), 1 μl reverse transcriptase M‐MLV (RNAse H‐free; 200 U/μl) and RNAse‐free dH_2_O were added to a final volume of 20 μl and the mixture was incubated for 60 min. at 42°C. Next, the reaction was inactivated by heating to 70°C for 15 min. Subsequently, 2 μl of the 20 μl reverse transcription reaction was subjected to PCR amplification using PCR Master Mix (cat. no. M7502, Promega, Madison, WI, USA) with initial denaturation at 95°C for 10 min., then 35 cycles of 94°C for 30 sec., 60°C for 30 sec. and 72°C for 45 sec. Products were quantified using a melting curve analysis. Amplification of GAPDH was performed to quantify PCR products and confirm the use of equal amounts of RNA. Relative gene expression was analysed using the 2^ΔΔCt^ method. Primer sequences (Shanghai Sangon Biotechnology Co. Ltd., Shanghai, China) of the rat genes were as follows: TIMP‐3 forward, 5′‐TCTGCAACTCCGACATCG‐3′, and reverse, 5′‐GCGTAGTGTTTGGACTGATAGC‐3′; aggrecan forward, 5′‐TCCGCTGGTCTGATGGACAC‐3′,and reverse, 5′‐CCAGATCATCACTACGCAGTCCTC‐3′;Type II collagen forward, 5′‐TCCTAAGGGTGCCAATGGTGA‐3′, and reverse, 5′‐GGACCAACTTTGCCTTGAGGAC‐3′; GAPDH forward, 5′‐CAAGTTCAACGGCACAGTCAAG‐3′, and reverse, 5′‐ACATACTCAGCACCAGCATCAC‐3′.

### Protein extraction and Western blotting

Whole cell lysates were prepared using RIPA buffer [50 mM Tris‐HCl (pH 7.4), 150 mM NaCl, 1% (g/ml) Triton X‐100, 0.1% (g/ml) sodium dodecyl sulphate (SDS), 2 mM ethylenediaminetetraacetic acid, 2 mM PMSF] in the presence of protease and phosphatase inhibitors. For the detection of p‐Smad3, Smad2/3, p‐ERK1/2 and ERK1/2 protein levels, equal amounts of total cellular extracts from rat chondrocytes were loaded on a 10% SDS‐polyacrylamide gel, subjected to electrophoresis and transferred to PVDF membranes by electroblotting. For TIMP‐3 protein levels, equal amounts of total cellular extracts were separated by 15% SDS‐polyacrylamide gel electrophoresis. For type II collagen protein levels, equal amounts of total cellular extracts were separated by 8% SDS‐polyacrylamide gel electrophoresis. Membranes were then blocked with 5% skim milk. Rabbit polyclonal anti‐TIMP‐3 (Abcam, Hong Kong), polyclonal anti‐rat aggrecan (Abbiotec, San Diego, CA, USA), Polyclonal anti‐rat type II collagen (Abcam), rabbit monoclonal anti‐p‐Smad3 (Abcam), rabbit polyclonal anti‐Smad2/3 (Novus, Littleton, CO, USA), rabbit monoclonal anti‐p‐ERK1/2 (Abcam) and rabbit polyclonal anti‐ERK1/2 (Cell Signaling Technology Inc., Danvers, MA, USA) antibodies were incubated with the membranes overnight at 4°C. The membranes were then washed and incubated with horseradish peroxidase‐conjugated donkey anti‐rabbit immunoglobulin G secondary antibody (Santa Cruz Biotechnology, Santa Cruz, CA, USA) at room temperature for 2 hrs. Finally, protein bands were visualized by chemiluminescence (Millipore, Billerica, MA, USA) using the Tanon 4200 SF Imaging Analysis system (Tanon Science and Technology Co. Ltd., Shanghai, China). The isolation of nuclear proteins was performed with the Nuclear Extract Kit (Keygen Biotech, NanJing, China) following the manufacturer's instructions, and equal amounts of total nuclear extracts were subjected to Western blotting analysis. Western blots were reprobed with monoclonal anti‐GAPDH antibody (Cell Signaling Technology Inc.) or a polyclonal anti‐rabbit lamin B1 (Sigma‐Aldrich, St. Louis, MO, USA) as loading controls. Intensity levels were quantified using Total Lab 100 software (Nonlinear Dynamics, Durham, NC, USA).

### Statistical analysis

All experiments were performed three times, and the results are expressed as the mean ± standard deviation. The significance of differences between groups was assessed using the Kruskal–Wallis analysis of variance test using SPSS 13.0 statistical software (SPSS, Chicago, IL, USA). Differences with a *P*‐value of <0.05 were considered statistically significant. _**_ = a *P*‐value of <0.01, NS = no significant difference.

## Results

### ERK1 siRNA2 and ERK2 siRNA2 specifically target ERK1 and ERK2, respectively

**Figure 1:**
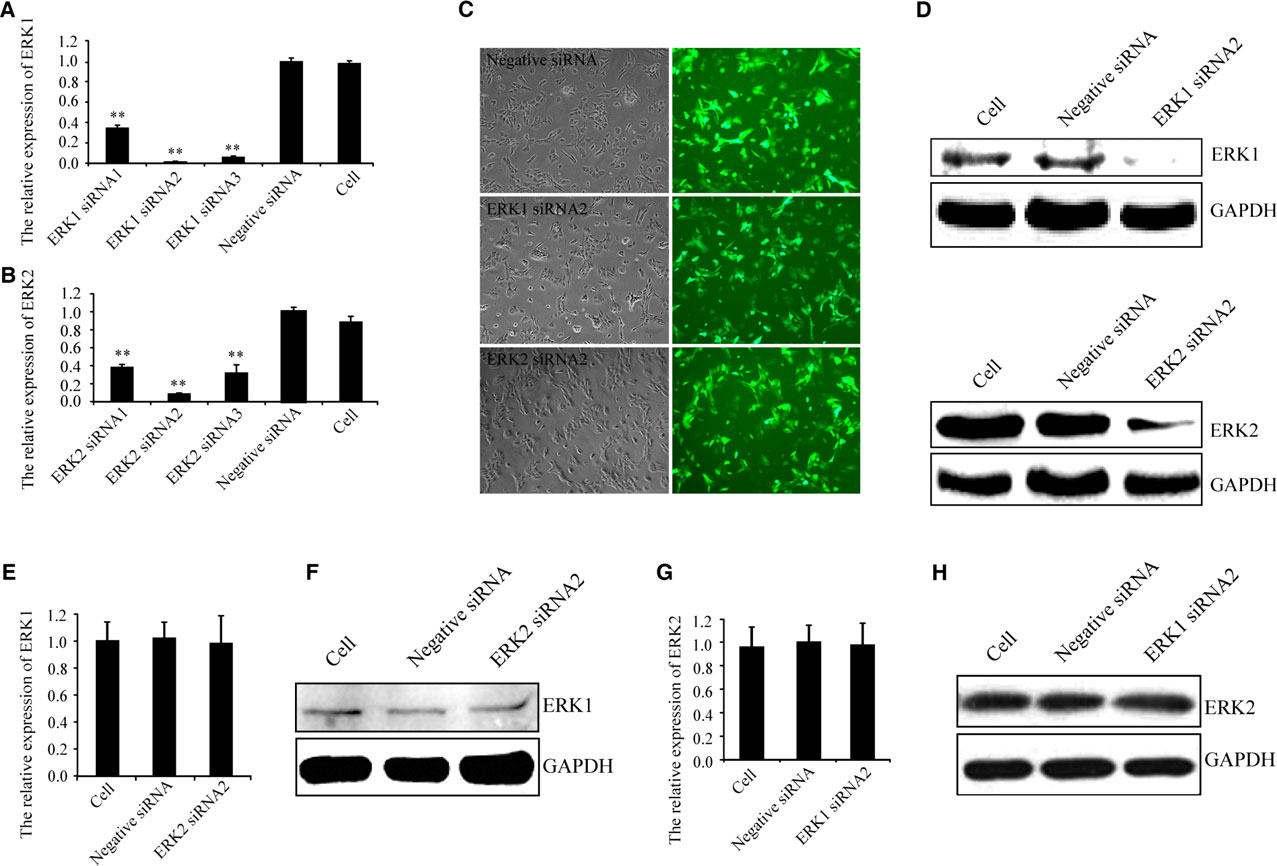
LV expressing ERK1 siRNA2 and ERK2 siRNA2 specifically target ERK1 and ERK2, respectively. (**A** and **B**) Real‐time PCR indicated that LV expressing ERK1 siRNA2 and ERK2 siRNA2 were the most efficient at targeting ERK1 and ERK2, respectively. (**C**) GFP expression demonstrated the high infection efficiencies of LV expressing negative siRNA, ERK1 siRNA2 and ERK2 siRNA2 after 96 hrs infection using fluorescence microscopy [light microscopy (left) and fluorescence microscopy (right), magnification = 100]. (**D**)And Western blotting indicated LV expressing ERK1 siRNA2 and ERK2 siRNA2 reduced ERK1 and ERK2 expression, respectively. Real‐time PCR and Western blotting indicated (**E** and **F**) ERK1 expression by ERK2 knock‐down and (**G** and **H**) ERK2 expression by ERK1 knock‐down. Data shown are from one representative experiment out of the three performed. ** *P*‐value of <0.01.

As shown in Figure 1(A and B), real‐time PCR revealed that the LV expressing ERK1 siRNA1, 2, 3 and ERK2 siRNA1, 2, 3 significantly reduced ERK1 and ERK2 expression relative to control cells. LV expressing ERK1 siRNA2 and ERK2 siRNA2 were the most efficient, leading to a 96.4% and 79.9% reduction in ERK1 and ERK2 expression, respectively. The negative siRNA had negligible effects on ERK1 and ERK2 expression. Chondrocytes were then infected with LV expressing ERK1 siRNA2 or ERK2 siRNA2. As shown in Figure 1(C), the expression of GFP 96 hrs after infection suggested high infection efficiencies of the LVs expressing negative siRNA, ERK1 siRNA2 and ERK2 siRNA2 in chondrocytes. Western blotting indicated that LV expressing ERK1 siRNA2 and ERK2 siRNA2 reduced ERK1 and ERK2 expression, respectively (Fig. 1D). Moreover, as shown in Figure 1(E–H), real‐time PCR and Western blotting revealed that LV expressing ERK2 siRNA2 had negligible effects on ERK1 or vice versa. Therefore, ERK1 siRNA2 and ERK2 siRNA2 were used to study the function of specific ERK isoforms.

### ERK1 knock‐down inhibits TGF‐β1‐induced TIMP‐3 expression

**Figure 2:**
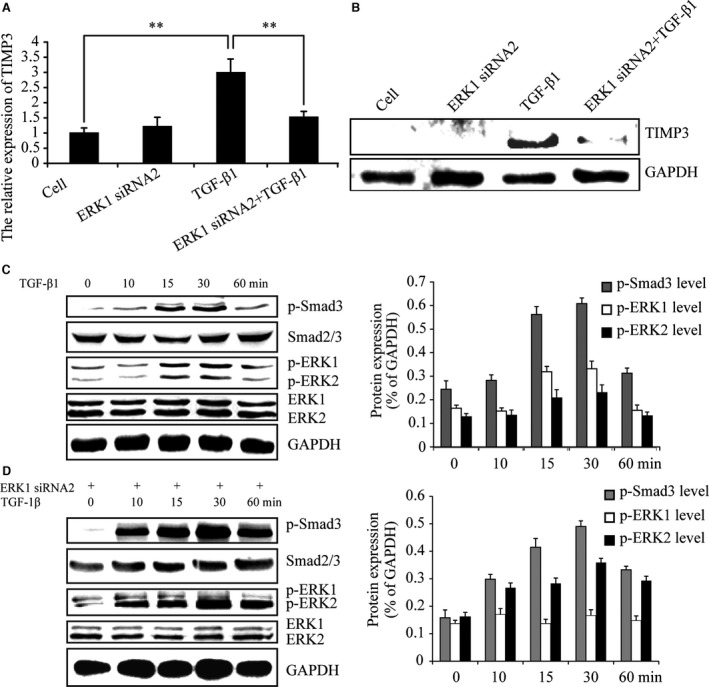
ERK1 knock‐down inhibits TGF‐β1‐induced TIMP‐3. (**A** and **B**) Real‐time PCR and Western blotting indicated TGF‐β1‐induced TIMP‐3 expression was significantly suppressed by ERK1 knock‐down. (**C**)At the indicated time‐points, Western blotting indicated ERK1/2 and Smad3 were phosphorylated after treatment with TGF‐β1. (**D**) At the indicated time‐points, Western blotting revealed that p‐ERK1 was inhibited in ERK1 siRNA2 expressing cells following TGF‐β1 stimulation. The intensity levels of p‐Smad3, p‐ERK1 and p‐ERK2 were quantified. Data shown are from one representative experiment out of the three performed. ** *P*‐value of <0.01.

As shown in Figure 2(A and B), real‐time PCR and Western blotting revealed TGF‐β1‐induced TIMP‐3 expression was significantly suppressed by ERK1 knock‐down. ERK1/2 and Smad3 were phosphorylated after TGF‐β1 treatment for 15 and 30 min. (Fig. 2C). These observations suggest that both the ERK1/2 and Smad2/3 signalling pathways are involved in TGF‐β1‐stimulated TIMP‐3 expression. Next, we assessed the role of ERK1 in TGF‐β1‐induced TIMP‐3 expression. As shown in Figure 2(D), the results confirm that ERK1 phosphorylation was inhibited by LV expressing ERK1 siRNA2 following TGF‐β1 stimulation. However, ERK1 knock‐down did not block the phosphorylation of ERK2 or Smad3. Together, these results suggest that ERK1 may be involved in TGF‐β1‐induced TIMP‐3 expression.

### ERK1 knock‐down reduced p‐Smad3 levels

**Figure 3:**
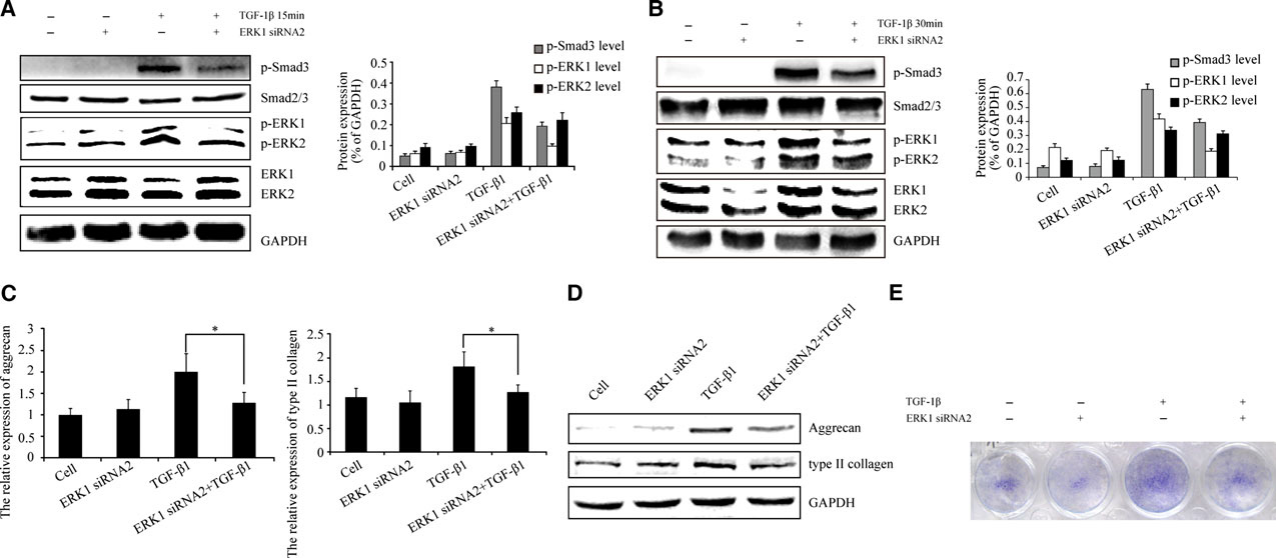
ERK1 inhibition reduces the TGF‐β‐Smad2/3 signalling. (**A** and **B**) Western blotting confirmed the quantity of p‐Smad3 was attenuated after TGF‐β1 stimulated for 15 and 30 min. when ERK1 was inhibited. (**C** and **D**) The real‐time PCR and Western blotting indicated TGF‐β1‐induced aggrecan and type II collagen expression wer significantly suppressed by ERK1 knock‐down. (**E**) Moreover, TGF‐β1‐induced intensity of toluidine blue staining of matrix was also decreased by ERK1 knock‐down. The intensity levels of p‐Smad3, p‐ERK1 and p‐ERK2 were quantified. Data shown are from one representative experiment out of the three performed. * *P*‐value of <0.05.

Although ERK1 knock‐down did not block Smad3 phosphorylation following TGF‐β1 stimulation (Fig. 2D), we investigated whether there existed quantity changes of Smad3 phosphorylation in whole chondrocyte extracts by ERK1 knock‐down. Chondrocytes were infected with or without LV expressing ERK1 siRNA2 for 96 hrs and then stimulated with TGF‐β1 for 15 and 30 minas Smad3 showed significant phosphorylation after 15 and 30 min.’ TGF‐β1 treatments (Fig. 2C). As shown in Figure 3(A and B), the data suggested that p‐Smad3 levels were attenuated when ERK1 was silenced. These data suggest that inhibition of ERK1 causes a reduction in TGF‐β1‐induced TIMP‐3 expression via suppression of the Smad2/3 signalling pathway.

Aggrecan and type II collagen, the target genes of TGF‐β‐Smad2/3 signalling, were examined to test the TGF‐β‐Smad2/3 signalling reduction by ERK1 knock‐down. As shown in Figure 3(C and D), real‐time PCR and Western blotting revealed TGF‐β1‐induced aggrecan and type II collagen expression were significantly suppressed by ERK1 knock‐down. Additionally, TGF‐β1‐induced intensity of toluidine blue staining of matrix was also decreased by ERK1 knock‐down (Fig. 3E). These data suggest that ERK1 knock‐down may reduce TGF‐β‐Smad2/3 signalling.

### ERK2 knock‐down has no effects on TGF‐β1‐induced TIMP‐3 expression

**Figure 4:**
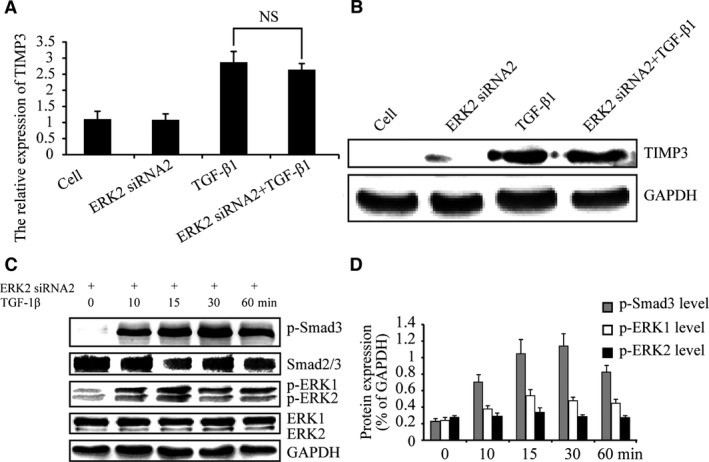
Inhibition of ERK2 has no effect on TGF‐β1‐induced TIMP‐3 expression. (**A** and **B**) Real‐time PCR and Western blotting indicated ERK2 knock‐down had no effect on TGF‐β1‐induced TIMP‐3 expression (NS = no significant difference). (**C** and **D**) At 0‐, 10‐, 15‐, 30‐ and 60‐min. time‐points, Western blotting revealed that p‐ERK2 was inhibited by LV expressing ERK2 siRNA2 following TGF‐β1 stimulation. The intensity levels of p‐Smad3, p‐ERK1 and p‐ERK2 were quantified. Data shown are from one representative experiment out of the three performed.

A set of experiments were undertaken to examine TIMP‐3 expression after TGF‐β1 stimulation by ERK2 knock‐down. Interestingly, the data indicated that TGF‐β1‐induced TIMP‐3 expression was not affected by ERK2 knock‐down (Fig. 4A and B). As shown in Figure 4(C and D), the results confirmed that the phosphorylation of ERK2 was inhibited by LV expressing ERK2 siRNA2 following TGF‐β1 stimulation. However, the levels of phosphorylated ERK1 and Smad3 were not altered. Taken together, these data suggest that ERK2 may have no effect on TGF‐β1‐induced TIMP‐3 expression. ERK2 siRNA2 induced a weak band for TIMP3 in condition without exogenous TGF‐β1 compared to control cell (Fig. 4B),which suggest that ERK2 may have an inhibitory role in endogenous TGF‐β signalling and TIMP3 expression.

### ERK2 knock‐down has no effects on p‐Smad3 levels

**Figure 5:**
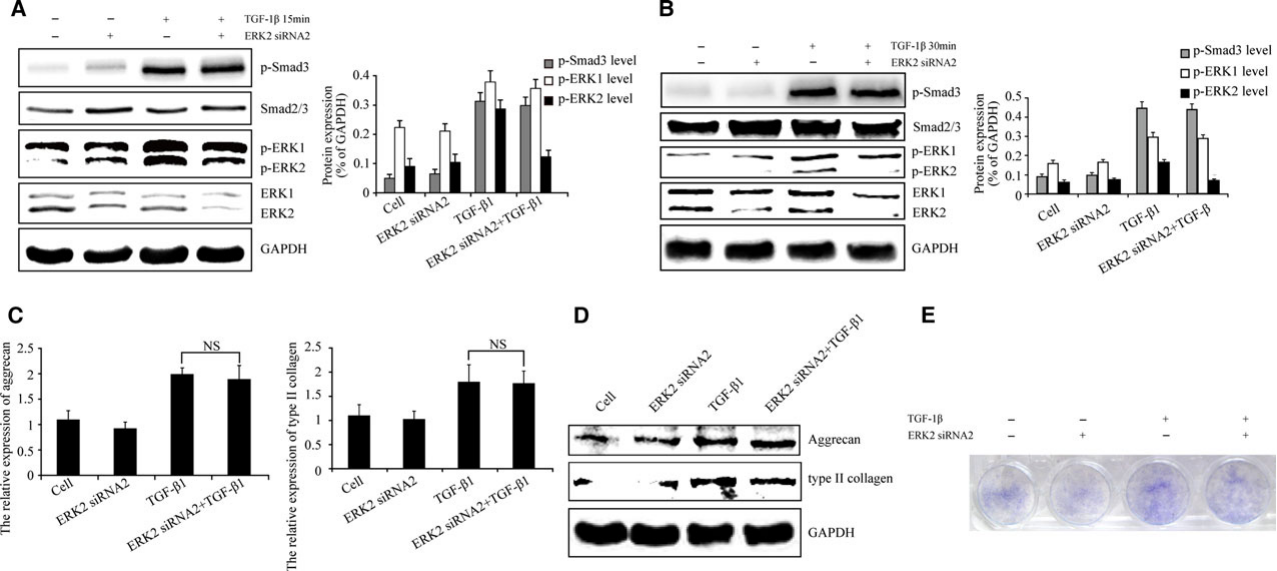
ERK2 knock‐down has no effect on the TGF‐β‐Smad2/3 signalling. (**A** and **B**) Western blotting confirmed knock‐down of ERK2 has no effect on p‐Smad3 levels after TGF‐β1 stimulated for 15 and 30 min. (**C** and **D**) The results indicated ERK2 knock‐down had no effect on TGF‐β1‐induced aggrecan and type II collagen expression. Moreover, (**E**) ERK2 knock‐down had no effect on TGF‐β1‐induced intensity of toluidine blue staining of matrix. The intensity levels of p‐Smad3, p‐ERK1 and p‐ERK2 were quantified. Data shown are from one representative experiment out of the three performed.

Next, we investigated whether p‐Smad3 levels in whole chondrocyte extracts were affected by ERK2 inhibition. Chondrocytes were infected with or without LV expressing ERK2 siRNA2 for 96 hrs and then stimulated with TGF‐β1 for 15 and 30 min. The results indicated no differences in p‐Smad3 levels by ERK2 knock‐down (Fig. 5A and B). These data suggest that ERK2 does not regulate TGF‐β‐Smad2/3 signalling and has no effect on TGF‐β1‐induced TIMP‐3 expression. Aggrecan and type II collagen, the target genes of TGF‐β‐Smad2/3 signalling, were revealed by real‐time PCR and Western blotting. As shown in Figure 5(C and D), knock‐down of ERK2 showed no effect on TGF‐β1‐induced aggrecan and type II collagen expression and TGF‐β1‐induced intensity of toluidine blue staining of matrix (Fig. 5E). These results suggest that inhibition of ERK2 may have no effect on TGF‐β‐Smad2/3 signalling.

### Inhibition of ERK1 and ERK2 has different effects on the accumulation of nuclear p‐Smad3

**Figure 6:**
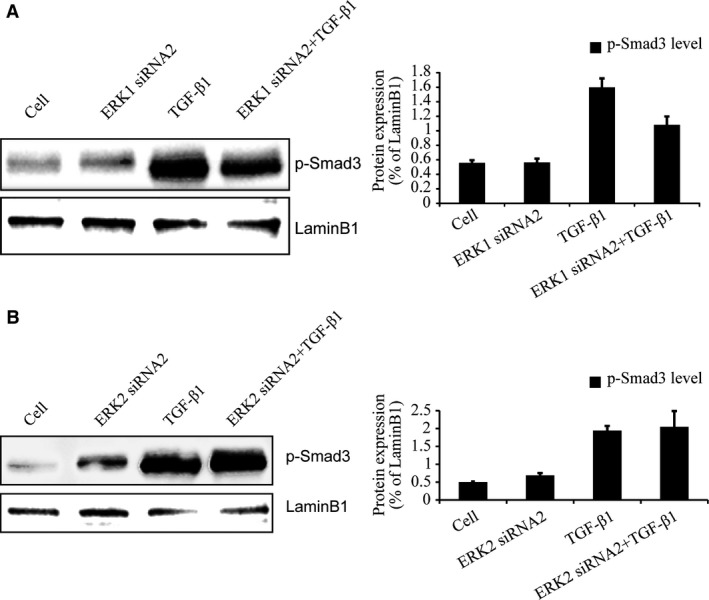
Inhibition of ERK1 but not ERK2 affects the accumulation of nuclear p‐Smad3. (**A** and **B**) Western blotting confirmed knock‐down of ERK1 reduced the accumulation of nuclear p‐Smad3 after TGF‐β1 stimulated for 30 min. However, knock‐down of ERK2 had no this effects. The intensity levels of p‐Smad3, p‐ERK1 and p‐ERK2 were quantified. Data shown are from one representative experiment out of the three performed.

Finally, nuclear p‐Smad3 levels were examined by Western blotting following ERK1 and ERK2 silencing because the nuclear accumulation of Smad2/3 may determine TGF‐β signalling. Chondrocytes were infected with LV expressing ERK1 siRNA2 for 96 hrs, and then stimulated with TGF‐β1 for 30 min. As shown in Figure 6(A), Western blotting indicated that nuclear p‐Smad3 level was reduced by knock‐down of ERK1. Knock‐down of ERK2 did not influence nuclear p‐Smad3 level (Fig. 6B). These results suggest that ERK1 promotes the accumulation of nuclear p‐Smad3, enhancing TGF‐β/Smad signalling while ERK2 has no such activity. As shown in Figure 6(B), in condition without TGF‐β1 treatment, ERK2 siRNA2 increased the band for nuclear‐accumulated p‐Smad3, which suggest that ERK2 may have an inhibitory role in endogenous TGF‐β signalling expression.

## Discussion

Our previous study reported that ERK1/2 could activate the Smad2/3 signalling pathway and up‐regulated TGF‐β1‐induced TIMP‐3 expression 9. However, the respective roles of ERK1 and ERK2 in the crosstalk with the Smad2/3 signalling pathway remain unknown. This study aimed to examine the involvement of specific isoforms of ERK in crosstalk with Smad2/3. Our results showed that ERK1 knock‐down could reduce p‐Smad3 levels in whole cell extracts and could prevent TGF‐β1‐induced TIMP‐3 expression up‐regulation. In addition, inhibition of ERK1 reduced the accumulation of the nuclear p‐Smad3, which suggested the TGF‐β/Smad signalling attenuation. Inhibition of ERK2 had no such effects. The results herein defined distinct roles of ERK1 and ERK2 during TGF‐β/Smad signalling‐induced TIMP‐3 induction, thereby highlighting a new level of regulation of ECM homoeostasis in cartilage.

Osteoarthritis is characterized by progressive loss of ECM in cartilage. The MMP family members are the major enzymes that degrade the components of the ECM. TIMPs are natural endogenous inhibitors of MMPs, and TGF‐β‐induced TIMP‐3 is one of the primary antagonists of MMP activity in cartilage. Smad2/3 is the canonical intracellular mediators of TGF‐β signalling; activated Smad2/3 is involved in TGF‐β‐induced cell proliferation, differentiation and target gene expression 10, 11, 12, 13. It has been shown that Smad2/3 siRNA blocks TGF‐β signalling and maintains TIMP‐1 expression in endothelial cells 14. Our results indicate that Smad3 phosphorylation is associated with increased TIMP‐3 expression in chondrocytes. These findings are consistent with a previous study that reported Smad2/3 signalling to be critical for TGF‐β‐induced TIMP‐3 expression in human chondrocytes 4.

Along with the canonical Smad signalling pathway, there are other non‐Smad‐mediated pathways downstream of TGF‐β, such as ERK1/2. The MAPK/ERK signalling pathway is required for nucleus pulposus‐derived mesenchymal stem cell differentiation and ECM biosynthesis 15. Inhibition of ERK phosphorylation blocks TGF‐β1‐induced human colon cancer cell migration 16. In chondrocytes, TGF‐β1‐induced TIMP‐4 expression is mediated partially by the ERK pathway and the transcription factor Sp1 17. In the present study, we demonstrated that ERK1/2 was involved in TGF‐β1‐stimulated TIMP‐3 expression, which is consistent with previous studies 8. We further demonstrated that ERK1 knock‐down inhibited TGF‐β1‐induced TIMP‐3 expression, while knock‐down of ERK2 had no effect on TGF‐β1‐induced TIMP‐3 expression.

Furthermore, crosstalk between ERK1/2 and Smad2/3 may control functional responses to TGF‐β. In fibroblasts exogenously stimulated with TGF‐β1, the ERK‐specific inhibitor PD98059 disrupted the Smad2/3/4 complex from translocating into the nucleus, blocking the expression and nuclear translocation of p‐Smad2C/L, indicating that ERK1/2 mediates Smad2/3 signal transduction 18, 19. The ERK1/2 pathway regulates various physiological events, including cell growth, differentiation and survival. The exact role of specific ERK isoforms in different cell types and among different cellular functions is poorly understood. In normal hepatocytes, ERK2 is required for epidermal growth factor‐dependent MAPK activation and cell proliferation 20. In rodent pain models, ERK2 is the key ERK isoform mediating nociceptive sensitization, while ERK1 plays a limited role in these processes 21. Conversely, other studies have suggested ERK1 activity is involved in hepatic stellate cell activation during liver fibrosis 22, 23. Increased ERK1 protein levels are associated with tumour progression and prognosis in gastric cancer 24. Additionally, a recent study suggested that both ERK1 and ERK2 were involved in the persistent efficient replication of enterovirus 71 25. Thus, the interactions between the specific ERK isoforms and Smad2/3 signalling are intricate and not yet fully understood. Owing to the therapeutic potential of TIMP‐3, it is important to fully understand the role of specific ERK isoforms in the regulation of the Smad2/3 signalling pathway in TGF‐β1‐induced TIMP‐3. In this study, RNA interference techniques were used to discriminate between the functions of ERK1 and ERK2. Our results indicate that while the phosphorylation of Smad3 is not blocked following ERK1 knock‐down, it is greatly reduced. However, our data also show that ERK2 has little influence on p‐Smad3 levels. These data suggested that ERK1 but not ERK2 may regulate TGF‐β‐Smad2/3 signalling. Aggrecan and type II collagen are the target genes of TGF‐β‐Smad2/3 signalling 26, 27, 28. And, TGF‐β‐Smad2/3 signalling is involved in increasing intensity of extracellular matrix 29, 30. Our results showed TGF‐β1‐induced aggrecan and type II collagen expression and intensity of matrix were significantly suppressed by ERK1 knock‐down instead of ERK2 knock‐down. Together, these data suggest that ERK1 instead of ERK2 is involved in the regulation of Smad2/3 signalling in TGF‐β1‐induced TIMP‐3 expression.

TGF‐β1 binds to type I and type II receptors and transduces intracellular signals through Smad2/3 31, 32. Phosphorylated Smad2/3 translocate into the nucleus and bind to transcriptional factors, regulating the transcription of target genes 33. The enhanced nuclear accumulation of Smad2/3 may promote TGF‐β signalling 34, 35. Therefore, we further investigated the level of nuclear p‐Smad3 following knock‐down of ERK1 and ERK2. The results suggested the accumulation of the nuclear p‐Smad3 was reduced following knock‐down of ERK1, but not ERK2. Together, these findings reveal possible novel mechanisms through which ERK1 cooperatively crosstalks with Smad2/3 to regulate TIMP‐3 expression. Nuclear shuttling, ubiquitylation and deubiquitylation, degradation, and phosphorylation and dephosphorylation of Smad are methods of regulating the nuclear retention of Smad 36, 37, 38, 39. The mechanism by which knock‐down of ERK1 reduces the accumulation of the nuclear p‐Smad3 requires further study.

In the present study, we found that ERK2 may have an inhibitory role in endogenous TGF‐β signalling and TIMP3 expression. We would focus on the role of ERK1/2 in endogenous TGF‐β signalling and TIMP3 expression in the further study. Also, we would like to test more target genes of TGF‐β‐Smad2/3 signalling and their crosstalks with isoforms of ERK. And 9xCAGA luciferase construct, as TGF‐β signalling reporter, would be tested to observe the ERK1 silencing to attenuate the activity.

In conclusion, our results elucidate the mechanism through which specific ERK isoforms regulate TGF‐β‐induced TIMP‐3 expression in rat chondrocytes. We demonstrated that knock‐down of ERK1 instead of ERK2 reduced p‐Smad3 levels in whole cell extracts and the nuclear accumulation of p‐Smad3, which may suggest crosstalk between ERK1 and Smad2/3 in the regulation of TGF‐β signalling. This study provides new evidence supporting the role of ERK1 in the regulation of ECM homoeostasis in cartilage.

## Funding

This work was supported by the National Natural Science Foundation of China (81101380) and Shanghai Ninth People's Hospital, Shanghai Jiao Tong University School of Medicine (syz2015‐008).

## Conflict of interest

The authors declare no conflict of interest in this work.
